# Genome Sequence of *Vibrio parahaemolyticus* VP103 Strain Isolated from Shrimp in Malaysia

**DOI:** 10.3389/fmicb.2016.01496

**Published:** 2016-09-21

**Authors:** Vengadesh Letchumanan, Hooi-Leng Ser, Kok-Gan Chan, Bey-Hing Goh, Learn-Han Lee

**Affiliations:** ^1^Division of Genetics and Molecular Biology, Faculty of Science, Institute of Biological Sciences, University of MalayaKuala Lumpur, Malaysia; ^2^Novel Bacteria and Drug Discovery Research Group, School of Pharmacy, Monash University MalaysiaBandar Sunway, Malaysia; ^3^Center of Health Outcomes Research and Therapeutic Safety, School of Pharmaceutical Sciences, University of PhayaoPhayao, Thailand

**Keywords:** *Vibrio parahaemolyticus*, seafood, genome, *Penaeus indicus*, antibiotic resistance

## Background

*Vibrio parahaemolyticus* is a Gram-negative bacterium that widely inhabits the marine and estuarine environments worldwide (Letchumanan et al., 2014). While the majority of strains isolated from environmental sources are innocuous members of marine microbiota, a small number of *V. parahaemolyticus* strains is capable of causing human illness and often associated with food borne gastroenteritis or diarrhea (Hazen et al., 2015; Raghunath, 2015). This organism has caused the highest number of seafood associated gastroenteritis cases in many countries including United States and Asian countries (Scallan et al., 2011; Newton et al., 2012).

In addition, there have been many reports of multidrug antibiotic resistance in *V. parahaemolyticus* worldwide (Odeyemi and Stratev, 2016). Our dependence on antibiotics to control this bacterial infections in humans, aquaculture, agriculture, veterinary medicine, and clinical setting has resulted in indiscriminate use which in turn led to the emergence of multidrug resistant strains in the biosphere (Letchumanan et al., 2015b, 2016a; Rao and Lalitha, 2015). Multidrug resistant *V. parahaemolyticus* strains have been isolated and detected from shrimp in Thailand (Yano et al., 2014), Malaysia (Al-Othrubi et al., 2011; Sani et al., 2013; Letchumanan et al., 2015a,c), and China (Peng et al., 2010; Xu et al., 2014). Resistance toward clinically used antibiotics will eventually hamper the treatment of bacterial infections in humans and potentially increase the fatality rate (Daniels et al., 2000). Therefore, monitoring *Vibrio* species in aquaculture surroundings is crucial for both human health and the aquaculture industry.

In order to gain better understanding of the multidrug resistance pattern, we studied the genome sequence of *V. parahaemolyticus* VP103 strain which was isolated from our previous study (Letchumanan et al., 2015a). *V. parahaemolyticus* VP103 strain was isolated from *Penaeus indicus* (Banana prawn) and originated from a fishery market in Malaysia. This strain exhibited multidrug resistance profiles toward 5/14 antibiotics tested. Based on the antibiotic susceptibility phenotype, the strain exhibited multiple-antibiotic resistance toward ampicillin, 3rd generation cephalosporins (cefotaxime and ceftazidime), and aminoglycosides (amikacin and kanamycin) (Letchumanan et al., 2015a).

This is a worrying situation as the antibiotic resistant profiles shown by *V. parahaemolyticus* VP103 include the recommended antimicrobial agents used in treatment of *Vibrio* spp. infections, including 3rd generation cephalosporin, fluoroquinolones, aminoglycosides, tetracycline, gentamicin, trimethoprim/sulfamethoxazole (Daniels and Shafaie, 2000; Shaw et al., 2014). Therefore, the whole genome sequence of *V. parahaemolyticus* VP103 was studied with respect to the multidrug resistance profiles to gain a better understanding of the antibiotic resistant patterns. The availability of this genome sequence of *V. parahaemolyticus* VP103 will aid as a basis for further in-depth analysis of the antibiotic resistance profile of *V. parahaemolyticus*.

## Materials and methods

### Genome sequencing and assembly and annotation

Genomic DNA of VP103 was extracted using Masterpure™ DNA purification kit (Epicenter, Illumina Inc, Madison, WI, USA) followed by RNase (Qiagen, USA) treatment (Ser et al., 2015; Letchumanan et al., 2016b). The DNA quality was quantified using NanoDrop spectrophotometer (Thermo Scientific, Waltham, MA, USA), and a Qubit version 2.0 fluorometer (Life Technologies, Carlsbad, CA, USA). Illumina sequencing library of genomic DNA was prepared using Nextera™ DNA Sample Preparation kit (Illumina, San Diego, CA, USA) and library quality was validated by a Bioanalyzer 2100 high sensitivity DNA kit (Agilent Technologies, Palo Alto, CA) prior to sequencing. The genome of VP103 strain was sequenced on MiSeq platform with MiSeq Reagent Kit 2 (2 × 250 bp Illumina Inc, San Diego, CA, USA). The trimmed sequences were *de novo* assembled with CLC Genomic Workbench version 5.1 (CLC Bio, Denmark).

### Genome annotation

Gene prediction was carried out using Prodigal 2.6, while rRNA and tRNA were analyzed using RNAmmer and tRNAscan SE version 1.21 (Lowe and Eddy, 1997; Lagesen et al., 2007; Hyatt et al., 2010). Gene prediction and annotation were performed using Rapid Annotation Search Tool (RAST, Aziz et al., 2008). Antibiotic resistance genes were analyzed using antibiotic resistance genes-ANNOTation (ARG-ANNOT, Gupta et al., 2014).

## Results

### Genome characteristics

The genome of *V. parahaemolyticus* VP103 consists of 4,988,425 bp with a mean genome coverage of 177.8-fold and with an average G + C content of 53.37% (Table 1). A total of 4820 genes was predicted of which 4648 genes were identified as protein coding genes. There are 91 RNA genes consisting of 10 rRNAs and 81 tRNAs. This genome sequence data of VP103 strain sequenced under this study has been deposited in DDBJ/EMBL/GenBank under Accession No. LBDB00000000. The version described in this paper is the first version, LBDB01000000. The genome sequences data are available in FASTA, annotated GenBank flat file, graphical, and ASN.1 formats.

**Table 1:** **General features of *Vibrio parahaemolyticus* VP103 genome**.

	**Vibrio parahaemolyticus VP103**
Genome size (bp)	4,988,425
Contigs	180
Contigs N_50_ (bp)	508,838
G + C content %	53.37
Protein coding genes	4648
RNA genes	91
rRNA	10
tRNA	81

### Virulence and antimicrobial resistance genes

**Figure 1:**
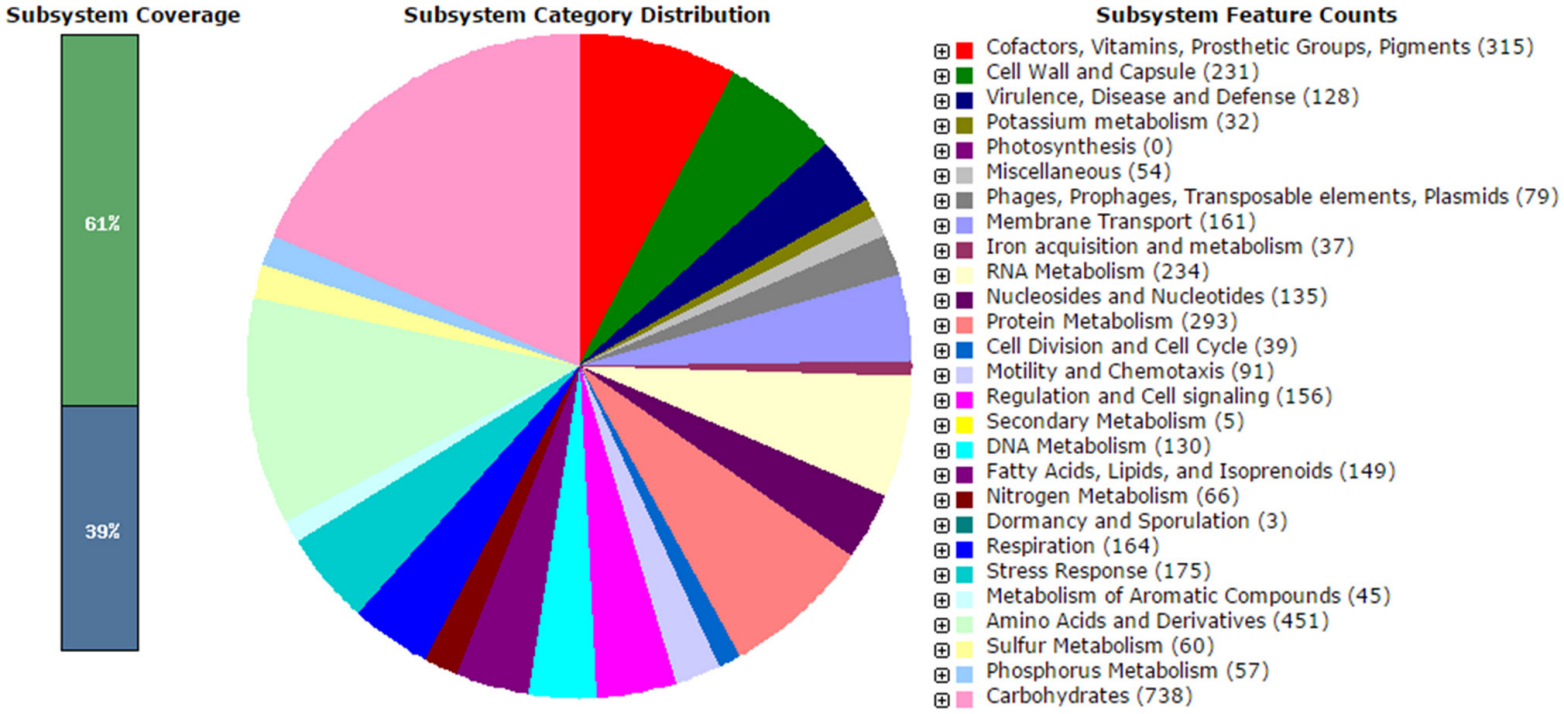
**Subsystem category distribution of *Vibrio parahaemolyticus* VP103 (based on RAST annotation server)**.

The analysis obtained from RAST server revealed 573 subsystems (Figure 1). The annotated genome has 97 genes responsible for resistance to antibiotic and toxic compounds including 19 genes for multidrug resistance efflux pumps, 4 genes for Beta-lactamase, 4 genes for multiple antibiotic resistance MAR locus, and 2 genes for aminoglycosides adenylyltransferase. The hemolysin gene was present in *V. parahaemolyticus* VP103 strain genome. The genome analysis on ARG-ANNOT noted the presences of β-lactam resistant gene, *bla* gene within the genome at 99% similarities when compared to other *V. parahaemolyticus* strains. The phenotypic resistance shown by *V. parahaemolyticus* VP103 toward ampicillin, cefotaxime, and ceftazidime is closely related to the gene coding Beta-lactamase in the genome. The gene coding aminoglycosides adenylyltransferase of *V. parahaemolyticus* VP103 confers resistance phenotypic observed toward amikacin and kanamycin.

Multidrug resistance profile seen in the phenotype and genes of *V. parahaemolyticus* VP103 genome illustrates how extensive antibiotics have been used in the aquaculture. Although antibiotics namely oxytetracycline, tetracycline, quinolone, sulphonamides, and trimethoprim are allowed in the Asian aquaculture industry (Rico et al., 2012; Yano et al., 2014), the extensive use of these antimicrobials has led to emergence of multidrug resistant strains in the environment. As the efficiency of clinical antibiotics has declined, the extensive use of antibiotics in the aquaculture and humans are in distress conditions due to spread of multidrug resistant strains (Letchumanan et al., 2015b). This situation is a definite cause of concern and warrants more stringent surveillance in the use of antibiotics. In summary, the whole genome sequence of *V. parahaemolyticus* VP103 will be useful in future studies to determine antimicrobial resistance and virulence attributes as well as mechanisms that enhance its environmental or host fitness.

## Author contributions

The experiments, data analysis and manuscript writing were performed by VL and HS, while KC, BG, and LL provided vital guidance and technical support. LL founded the research project.

### Conflict of interest statement

The authors declare that the research was conducted in the absence of any commercial or financial relationships that could be construed as a potential conflict of interest.
